# Centering autonomy and choice to support oral PrEP utilization among people who inject drugs: qualitative lessons from HPTN 094 INTEGRA

**DOI:** 10.1186/s13722-024-00520-3

**Published:** 2024-12-18

**Authors:** Amaya Perez-Brumer, Rose Schmidt, Rebecca Kennedy, Jordan E. Lake, Yolanda R. Villarreal, Sydney Bornstein, Irene Kuo, Omar Nieto, Julie Franks, Cecile Denis, Nabila El-Bassel, Steve Shoptaw, Peter Davidson, Laramie R. Smith

**Affiliations:** 1 Dalla Lana School of Public Health, University of Toronto, Toronto, Canada; 2 Institute of Mental Health Policy Research, Centre for Addiction and mental health, Toronto, Canada; 3 Division of Infectious Diseases and Global Public Health, University of California, San Diego, San Diego, CA USA; 4 Department of Internal Medicine, University of Texas Health Science Center at Houston, McGovern Medical School, Houston, TX USA; 5 Department of Family and Community Medicine, University of Texas Health Science Center at Houston, McGovern Medical School, Houston, TX USA; 6 George Washington University Milken School of Public Health, Washington, DC USA; 7 Department of Family Medicine, University of California, Los Angeles, CA United States of America; 8 Columbia University, ICAP Mailman School of Public Health, New York, NY USA; 9 Perelman School of Medicine, Department of Psychiatry, University of Pennsylvania, Philadelphia, PA USA; 10 School of Social Work, Columbia University, New York, NY USA; 11 Department of Family Medicine, University of California, Los Angeles, Los Angeles, CA USA

**Keywords:** Pre-exposure prophylaxis, PrEP, People who inject drugs, Integrated care, Qualitative research, HIV prevention trials network

## Abstract

**Background:**

Oral Pre-exposure prophylaxis (PrEP) is effective in preventing HIV transmission. However, despite high rates of HIV risk behaviors among people who inject drugs (PWID), this population remains underserved by current HIV prevention efforts in the United States. To address this challenge, we conducted an in-depth exploration of perspectives on using oral PrEP among PWID engaged in the HIV Prevention Trials Network (HPTN) 094 INTEGRA Study.

**Methods:**

Guided by the Practical, Robust, Implementation, and Sustainability Model (PRISM), our qualitative study drew on semi-structured interviews conducted as part of the embedded implementation science evaluation of HPTN 094 INTEGRA. Seventy-seven PWID participants from five sites across New York City, Houston, Los Angeles, Philadelphia, and Washington DC were interviewed to assess intervention delivery, care access, and engagement sustainability. Audio files were transcribed verbatim and analyzed via an inductive and deductive thematic approach.

**Results:**

Most participants (*n* = 46, 59.7%) discussed oral PrEP during their interview, though not directly prompted. Participants discussing PrEP had a mean age of 41.6 years and were predominantly white (54.3%) and cisgender men (60.9%). Among these, 15 participants described using PrEP. All participants had facilitated access to oral PrEP. Yet, the choice to use PrEP was influenced by personal risk perceptions, (mis)information about PrEP, and external factors (i.e. housing, financial security), which, for some, limited the autonomy to use PrEP. Two key themes emerged among participants using PrEP: ease of access and perceptions of high HIV risk. Those not using PrEP described two themes: low risk perception and prioritizing more urgent needs. Among participants not using PrEP a subgroup commonly described ambivalent interest, PrEP knowledge gaps, and PrEP readiness (i.e., contemplation).

**Conclusions:**

Qualitative findings highlight that facilitated PrEP access was insufficient to motivate use for many participants. Rather, PrEP decision-making process (i.e., choice) was linked to risk perception and individuals’ capability to leverage PrEP as a resource based on their circumstances (i.e., autonomy). Participants’ descriptions of the centrality of choice and autonomy for PrEP use underscore that ease of access is a necessary pre-condition, but person-centered interventions should also address housing, financial stability, and urgent medical conditions to promote PrEP utilization among PWID.

**Clinical trial registration:**

NCT04804027.

## Introduction

Oral Pre-exposure prophylaxis (PrEP) is an effective HIV prevention strategy with the potential to reduce incident HIV infections among at-risk populations, including people who inject drugs (PWID) [1]. It is estimated that 1.46% of the US population injects drugs, yet PWID represent 7% of new HIV infections in the US [2]. Despite the disproportionate burden of HIV suggesting the need for PrEP, engagement in the PrEP care continuum among PWID remains low [3–5]. Complex factors influence PWID engagement in the PrEP care continuum, including limitaions in their ability to engage in independent decision-making (i.e., autonomy). For example, PWID experience stigma and discrimination in healthcare settings, which can be a significant barrier to accessing and/or being offered PrEP as a choice for HIV prevention [6–9]. Discrimination and implicit biases can impact providers’ willingness to prescribe PrEP to PWID [10, 11]. As a result, knowledge of PrEP among PWID may be low [12, 13], and PWID may hesitate to disclose information about their drug use and associated risk behaviours to healthcare providers [10, 11, 14].

Evidence suggests that PWID can successfully access and adhere to PrEP when services are tailored to their diverse needs [3, 10, 15–18]. Even when PrEP is accessed and initiated, factors such as navigating complex and segmented healthcare systems, accessing accurate and appropriate health information, scheduling and attending numerous appointments, and the geographic dispersion of various health services can limit PrEP persistence [18–21]. Unmet survival needs such as poverty, homelessness, and healthcare access to address other co-occurring health conditions can also limit the prioritizing of PrEP and/or disrupt PrEP care among PWID [11, 20, 22–25]. Further, the criminalization of drug possession and use can interrupt PrEP use and, in turn, inadvertently increase HIV incidence and mortality among PWID [3, 19, 26].

Integrated care models that address PWID’s complex needs, including HIV prevention and medications for opioid use disorder (MOUD), have been shown to optimize engagement in the HIV care continuum and support improved substance use treatment outcomes [18, 27–29]. For instance, interventions targeting PrEP uptake and adherence for PWID are highly acceptable for PWID when they are incorporated into community-based integrated care models at syringe service programs [24, 30, 31]. However, lived realities of PWID, such as the interplay of housing and economic instability, along with survival needs experienced by many PWID, can reduce PrEP interest [10, 25, 32] and limit the accessibility of integrated health services from traditional bricks and mortar clinics, when they exist [33].

### HPTN 094 INTEGRA study

The ongoing HPTN 094 INTEGRA Study is a controlled, individually randomized, two-arm open-label study that tests the efficacy of delivering integrated care delivered on a mobile unit combined with a navigation condition compared to a condition that uses navigation to direct participants to existing community-based services. The study intends to enroll 450 PWID living with HIV or at risk for HIV across five major cities in the United States (New York City, Houston, Los Angeles, Philadelphia, and Washington DC). This paper draws on interviews with participants in the ongoing INTEGRA Study to examine the complexities of PrEP utilization and active injection drug use despite having facilitated access to oral PrEP.

## Methods

### Implementation science (IS) framework and study design

As a hybrid type 1 effectiveness implementation RCT, data collected for this study was guided by the Practical, Robust, Implementation, and Sustainability Model (PRISM) [34] to assess facilitators and barriers of implementing integrated care through the HPTN 094 trial. Specific to this analysis, the PRISM specifies that the implementation and uptake of evidence-based interventions, such as PrEP and MOUD will be influenced by four theoretical determinants to characterize (i) the patient (i.e., PWID) and healthcare delivery (i.e., mobile medical unit) organizational perceptions of integrated evidence-based interventions, (ii) characteristics of PWID patients and the mobile medical unit that affect the delivery of integrated care, (iii) factors in the external community environment that can influence how integrated care was delivered by the mobile medical unit and accessed by PWID, and (iv) systems-level factors and infrastructure that affect or are affected by the delivery of integrated care through the mobile unit. This process prospectively examined the role these determinants had on the implementation process and outcomes during the conduct of the RCT (e.g., perceptions of oral PrEP in the context of facilitated access) and on subsequent observed clinical outcomes among PWID following the evaluation of the RCT (e.g., adoption and maintenance of ART/PrEP and MOUD at week 26 and 52 post-baseline, respectively).

Eligibility for the INTEGRA study included: at least 18 years of age; urine test positive for recent opioid use and evidence of recent injection drug use; diagnosis of OUD; willing to start MOUD; able and willing to give informed consent, provide adequate locator information and complete an Assessment of Understanding. For people not living with HIV, additional eligibility included self-reported sharing injection equipment and/or condomless sex with a partner with unknown or positive HIV status (past three months). The enrollment visit for both study arms includes provision of HIV counseling, PrEP information, and condoms. Subsequently, for intervention arm participants, PrEP oral pill regimens can be dispensed at the mobile health unit (emtricitabine/tenofovir disoproxil fumarate [FTC/TDF] or emtricitabine/tenofovir alafenamide [FTC/TAF]), or a prescription for another regimen can be provided for fulfillment at a pharmacy. Control arm participants are referred to brick and mortar facilities in the community for PrEP, Peer navigators provided support to participants in both study arms. Full trial protocol was approved for all sites by a central institutional review board (Advarra), additionally University of Toronto Research Ethics Board approval was obtained for secondary data analysis. All participants provide written informed consent.

### Semi-structured interview procedure

Data presented here are from the 46 interviews where participants discussed oral PrEP in interviews. These are a sub-sample of Implementation Science (IS) interviews that were conducted with PWID (*n* = 77) across the five study sites to elicit multi-level PRISM determinants (i.e., patient, healthcare delivery, community environment, structural) affecting the intervention delivery, care access, and ability to sustain engagement in both integrated care services and community-based services. All IS qualitative sub-study participants were recruited from HPTN 094, however, IS participants received separate participant IDs preventing assessment of whether participants were assigned to active control (i.e., PrEP access through brick-and-mortar services) or intervention (i.e., PrEP access through mobile van) for 26 weeks. IS interviews recruited participants during (61%) and post 26-weeks (39%) to further understand experiences accessing and transitioning off of integrated care or peer navigation only. The rationale for not linking IS data to participants’ clinical outcomes was to ensure that IS data could be analyzed before the completion of the RCT to inform and improve how the study was delivered in local communities. The findings presented here do not reflect the HPTN 094 clinical results as the trial is ongoing.

All participants invited to join the IS sub-study had consented to be re-contacted and provided written informed consent. Interviews lasted approximately 20–45 min and participants were compensated $50.00 for their time. All interviews were conducted in English, de-identified, audio recorded and transcribed verbatim and assigned a random numerical identifier. The interviews followed a semi-structured guide, which included questions about barriers and facilitators to accessing MOUD and HIV services; experience with the INTEGRA study; and possible impact of COVID-19 of services. Direct questions about PrEP were not included, rather questions and probes asked about HIV prevention and care services and, if PrEP was referenced, additional probes were included to illicit further details.

### Qualitative analysis

IS interview transcripts were analyzed using a pragmatic inductive and deductive thematic approach [35], involving mapping emergent themes onto the a priori domains aligned with the PRISM framework. During this primary analysis, the IS team identified emergent themes related to PrEP use that warranted a targeted sub-analysis. The analytic approach for sub-analysis involved numerous co-authors: RS coding all discussions of HIV (including PrEP, HIV treatment, testing and prevention strategies) in participants’ transcripts using MaxQDA data analysis software (www.maxqda.com); APB, RS and RF iteratively identify themes with illustrative quotations; and presenting and refining themes by the core qualitative investigative team until consensus was met between APB, RS, RF, LRS. Based on discussions, we categorized the coded segments by participant type: those describing PrEP use (*n* = 15), those who discussed PrEP but not PrEP use (*n* = 31), those with HIV and ineligible for PrEP (*n* = 5), and no PrEP discussion during the interview (*n* = 26). We excluded interviews where PrEP was not discussed (*n* = 26) and from participants with HIV who were not eligible for PrEP (*n* = 5). The analytic sample for the present analysis is drawn from 46 interviews.

## Results

Among the 46 participants who discussed PrEP in interviews, mean age was 41.6 years, and most participants were white (54.3%) and cisgender men (60.9%). See Table 1 for participants’ demographic characteristics.

**Table 1:** Demographic characteristics of implementation science interviews among HPTN 094 INTEGRA participants who discussed PrEP

		Total (*N* = 46)	
		*N*	%
**Site**			
	Los Angeles, CA	14	30.4%
	Houston, TX	10	21.7%
	Philadelphia, PA	9	19.6%
	New York City, NY	9	19.6%
	Washington DC	4	8.7%
**Race (Non-Hispanic)**			
	White	25	54.3%
	Black/ African American	9	19.6%
	More than one race	6	13.0%
	Latine/x	2	4.3%
	American Indian/ Alaskan Native	2	4.3%
	Not listed	2	4.3%
**Ethnicity**			
	Hispanic	18	39.1%
	Non-Hispanic	27	58.7%
	Not reported	1	2.2%
**Gender**			
	Cisgender Man	28	60.9%
	Cisgender Woman	17	37.0%
	Not listed	1	2.2%
**Age (Mean**,** SD)**		41.6 (11.3)	

Across participants oral PrEP utilization was described as part of other enacted HIV prevention strategies and influenced by the larger theme of autonomy and power to make decisions about one’s own health. Further, descriptions of urgent need to address structural factors in the external environment like unstable housing and precarious financial status, as well as characteristics of the patient related to urgent medical conditions and addiction treatment were described as factors restricting the ability to make autonomous decisions regarding PrEP. To detail the complexity between these determinants, results are presented among those using PrEP and among those who discussed PrEP but not report PrEP use. Two key themes were identified among PrEP users: the ease of access and perceived need for PrEP relative to perceived risk for HIV. Among participants discussing PrEP but not PrEP use, two themes were identified: perceptions of low risk for HIV and urgent needs prioritized over initiating PrEP. Notably, among participants who did not discuss PrEP use, a subgroup noted mixed and/or ambivalent interest in PrEP, among whom key themes identified included: PrEP knowledge gaps and PrEP readiness (i.e., contemplation).

### Among participants describing oral PrEP use

Among the 15 participants who described using PrEP, two main reasons were identified: PrEP was easy to access (healthcare organizational determinants), and they perceived themselves to be at increased risk for HIV and thus chose to use PrEP as an evidence-based strategy to prevent HIV acquisition and/or transmission. These themes were not mutually exclusive.

#### Ease of PrEP access

The most consistent theme as to why participants used PrEP was the ease of access to initiate taking PrEP. For example, the mobile unit was often described as facilitating access to PrEP due to proximity to where people resided. Furthermore, as noted by one participant, this ease of access allowed him to avoid “*getting caught up*” in street culture the way he might have if he had to travel further to an in-person medical clinic:

“I think it’s better for the mobile service because you got the doctor, you got everything right here– medication, whatever you want. They can prescribe it for you. If you get it or if you use it, you can get it right here. You don’t have to go out in the street. I don’t have to get caught up out there. I can just come right here to the mobile unit and get what I need and go back to my spot. […] It’s been easy.” (Los Angeles 60-year-old, Black, man).

The mobile unit also helped to address some of the specific barriers to accessing PrEP at traditional brick-and-mortar clinics, such as not having to arrange transportation and find alternatives for caretaking responsibilities. In addition to ease of access, several participants mentioned that the privacy and tailored services made them feel more respected by healthcare providers. The design of the integrated care intervention also provided participants with an opportunity to attend to multiple issues related to their substance use in one location:

“Instead of just talking about it, I’m actually doing things […] having all this stuff here and like in your face [….] so many things are offered. It’s like getting one thing accomplished, getting starting on Subs [MOUD], like getting the PrEP […] once I started getting one thing done, I start feeling good and it keeps going. It like steamrolls, snowballs. I keep going.” (Philadelphia 34-year-old, white, man).

While many participants using PrEP perceived themselves to be at heightened HIV-risk due to specific behaviours, as described in more detail below, for some participants the motivation for taking PrEP was not necessarily tied to a specific behaviour. Instead, these participants said they used PrEP because it had been offered to them, and it was easy to start PrEP as part of the study. These participants expressed having a *“just in case”* attitude when it came to using PrEP. While some of these participants perceived their personal risk of contracting HIV as low, they demonstrated a clear desire to protect themselves from HIV. For example, one participant noted that he took PrEP *“Just to be on the safe side”* (Los Angeles 40-year-old, white, man).

#### Perceptions of being at high risk for HIV transmission

Among participants that said they used PrEP, many did so because they recognized that they participated in activities that increased vulnerability to HIV. These participants described choosing PrEP to reduce potential HIV acquisition related to injecting drugs and engaging in higher risk sexual activities including sex work. For example, when asked why she started PrEP one participant responded:

“I have started the medication to help me prevent myself from getting HIV, so that was a very good thing because whenever I’ve had to in my life do other things to pay the bills or to get things done because that’s all I’ve known in my life, you know, selling my body was another part of the struggle. So that was something that also yes was very helpful because helping me prevent myself from getting HIV is very important especially with injecting and also the lifestyle.” (Houston 36-year-old, mixed race, woman).

This quote illustrates the complex risk environment some street involved PWID experience while engaging in survival sex work and injection drug use and her commitment to preventing HIV transmission. Furthermore, in another interview from Houston, while discussing PrEP use, a participant noted that he was engaging in behaviors that might transmit HIV, for example, “*Yeah… needle use. Unprotected sex”* (Houston 39-year-old white, man).

When probed with a follow up question asking if he would use new needles or share used needles he responded, *“whatever*,* whatever was available.”* He went on to explain that his daily injection drug use meant that sometimes he used drugs when new needles were not available to him because, “*it got stolen*,* or I broke it”* and that he would ask “*whoever was around”* regardless of whether the person was *“dirty”* [person living with HIV].

Even when engaging in harm reduction (e.g., using new needles) some participants described wanting to do all that they could to prevent HIV, which included taking PrEP. For example, when asked what his reasons were for wanting to start PrEP one participant responded:

“I’m an injection user, so there’s always sometimes a chance I might be using a used needle or something like that, not intentionally, but you never know.” (Los Angeles 36-year-old, white, man).

This illustrates how being able to choose PrEP as an option to HIV prevention facilitated participants being able to make decisions about managing HIV risk behaviours in the context of not having consistent access to harm reduction services and access to new injecting supplies.

### Among participants who describe not using PrEP

Three key themes were identified among the 31 participants who discussed PrEP but not PrEP use in their interviews including two themes related to the lack of need for PrEP (perceived themselves as low risk for HIV and having more pressing needs to attend to than HIV prevention), and another highlighting PrEP ambivalence.

#### Perceptions of low risk for HIV

##### Low sexual risk

Some participants felt no need for PrEP because they did not engage in specific sexual behaviours they considered at risk for HIV transmission. One participant, when asked if she had been offered any HIV related services responded, “*Yeah*,* absolutely*,* yes I was offered that medication*,* which I turned down because A*,* I wasn’t having sex*,* B I wasn’t engaging in any risky behaviors at this point*,* so I didn’t need to take it”* (Philadelphia 48-year-old, white, Hispanic, woman). Participants also described that due to their monogamous relationships there were at low risk for HIV transmission. When asked why he declined PrEP, one participant responded, *“Because I have a girlfriend and we’re committed*,* and we don’t cheat on each other”* (Los Angeles 44-year-old, white, man).

Other participants noted that PrEP was not necessary because they “*never had*,* well*,* much anal sex”* (Los Angeles 61-year-old, white man), or they only received oral sex. Some described sex work as associated with increased risk of HIV transmission due to having multiple sexual partners and possible engagement in unprotected sex with unknown partners. As described, *“I may have*,* did a lot of drugs but I wasn’t like ever in the streets. Like I never like*,* like sold my body or anything”* (Houston 24-year-old, white, Hispanic, woman).

Many people who had one partner also spoke about the importance of HIV testing, in conjunction with monogamy as a strategy for HIV prevention. For example, when asked if she accessed any HIV-related services she responded, “*Yeah. I’ve never really been concerned with HIV just because I’ve had one partner”* (Houston 27-year-old, white, woman). Later in the conversation she further explained, *“Like I said*,* we’ve been tested […] and I haven’t had sex with anybody new.*” Similarly, when asked why he declined PrEP, a participant from Washington DC said:

“Like I said, I’m not a person that run around and I never shared my needle with nobody. I didn’t run around with women. So I’ve been tested quite a few times. When I set up house, what I call set up house, when two people living together, that’s the first thing we do and I bring that up, ‘Look, you go get tested, I go get tested. If we’re going to be together, this is how it’s got to be.’” (Washington DC 67-year-old, Black, man).

As highlighted by the quotation above, multiple alternative prevention strategies were sometimes employed by people who did not use PrEP. This included not sharing injecting equipment, engaging in frequent HIV testing, and encouraging their partner to get tested for HIV before starting a new sexual relationship.

Another prominent HIV prevention strategy employed was the use of condoms: *“I mean*,* I don’t want to take no chances. So*,* I always use condoms anyway. So*,* I wouldn’t feel I need the PrEP”* (New York City 54-year-old, Black, man). The mobile unit was also cited as helping to facilitate access to condoms for these participants. For example, a 35-year-old Black man from Houston who was not interested in PrEP was asked what he did to prevent sexually transmitted infections. He stated, *“Oh*,* yeah. Yeah*,* I’ll get the condoms. They make sure I get some condoms”.*

##### Low injection risk

Among those who did not use PrEP, another commonly described HIV prevention strategy was not sharing needles and other injecting equipment. When asked about the services offered to her on the mobile unit, one participant stated, *“They were trying to put me on PrEP*,* but I really did not see the necessity. I don’t share”* (Washington DC 26-year-old, race not listed, Hispanic, woman). In addition to perceiving herself to generally be at low risk for HIV transmission, the participant was uninterested in using PrEP because she did not *“believe on going on medicine unless you actually need it.”*

While some participants cited not sharing injecting equipment as the reason for not using PrEP, there were often inconsistencies in some of these accounts. For example:

“Yeah, always clean needles. One time […] I went to the ‘hood to get some dope, and I left my needle. I asked the guy if he had some. He didn’t have any fresh ones. He gave me one, and I looked at it, and it was used, but I wanted it so bad I used what he had. Even then I’m like, ‘This is horrible. I feel disgusting.’ But I needed to get my sick off, I got checked right after that and I was clean, thank God.” (Houston 35-year-old, Black, man).

This quote underscored that the limited availability of sterile drug paraphernalia when participants want to use explains why they sometimes shared equipment. On the other hand, in different locations, participants noted that “*getting clean needles”* was ever a problem; one participant described the importance of other mobile harm reduction services in facilitating that access, *“No*,* not really. This area especially- the truck is up there right now*,* like it’s got the needles every day. It’s always available.”* (New York City 37-year-old, white, man).

Some participants said that they did not share injecting equipment because they always used alone. However, it was unclear from these participants if they used sterile equipment each time they injected. One participant stated that they have been asked multiple times on the mobile health delivery unit if they wanted to start taking PrEP, but they did not feel it was necessary because:

“I don’t live that kind of lifestyle to share needles. I never—it was always super-clean thing. It always a very private thing. It was never a social thing. Just the thought of me catching AIDS, it’d have to be total freak accident for me to have it—for it to happen. Not any of the activities that I’m partaking in.” (Los Angeles 38-year-old, white, gender not identified).

#### Urgent needs prioritized over initiating PrEP

A few participants described choosing to not use PrEP because they had more pressing medical or social concerns that took priority. For example, when asked if he had started taking PrEP, one participant said, *“I haven’t been as responsive as I’d like to be because I’ve just got so many problems to deal with”* (Los Angeles 23-year-old, white, man). Some of these pressing concerns included unstable housing, urgent medical conditions (e.g., wound care), and addiction treatment. When asked about HIV services and prevention, some participants noted, *“I don’t need that. Housing and supportive*,* like*,* medically assisted treatment services. Those two pieces”* (New York City 36-year-old, Black, man). Similarly:

“I’m looking into getting into a program, an opioid withdrawal program. That’s something I have to think about […] I’ll work with the services that are available here at the mobile unit when I’m ready […] because I have to focus mainly on my living situation, my housing. Once I get my housing, and I have a roof over my head, a permanent roof over my head, then I’ll probably look into going with whatever services are available at the mobile unit.” (Los Angeles 51-year-old, race not listed, Hispanic, man).

### PrEP ambivalence

Among the 31 participants who discussed PrEP but not PrEP use, 7 participants described mixed and/or ambivalent interest in PrEP. Among this subgroup, two themes were identified related to their perceived need for PrEP: PrEP knowledge gaps and interest in/readiness for future PrEP use (i.e., contemplation).

#### PrEP knowledge gaps

Some participants expressed opinions about PrEP suggesting misunderstandings about the medication and populations that could benefit from its use. Further, there was often overlap between participants who perceived themselves at low risk for HIV transmission and those who had knowledge gaps. For example, when asked if he was interested in PrEP, one participant responded:

“I am, yeah. I don’t know if I’d be— I mean, yeah, I think I would. I don’t know the side effects or anything. I don’t know enough about it. I know in the community, people think it’s more a thing for homosexual men to take, but I know it’s– being an IV user, you’re way more prone to HIV and stuff like that…. I’ve definitely heard that [about PrEP], yeah. But its not—for me, I don’t really care about that.” (Houston 30-year-old, American Indian/Alaska Native, Hispanic, man).

This participant acknowledged that PrEP was commonly perceived to be for gay men. Despite indicating a possible awareness of his vulnerability to HIV infection due to his injecting drug use, the participant ultimately did not see PrEP as something relevant for him.

Among some participants, the limited understanding of who PrEP was for was aligned with stigmatizing views of who they perceived would be at risk for HIV. For example, when asked what services he had used and not used on the mobile health delivery unit one participant recounted declined services around HIV testing and prevention because, *“I’m not going go around sharing needles*,* I’m not going to do things that are going to be stupid. So*,* I don’t think I have AIDS and I think I’m above that*,* to be honest.”* (Los Angeles 61-year-old, white, man).

Another knowledge gap was related to the fact that PrEP is designed for people who are currently HIV negative, serving as a pre-exposure preventive measure. For example, when asked what she did for HIV prevention, one participant noted, “*I did check that stuff […] But I didn’t have HIV though*,* I didn’t get that. But I do have some medicine*,* I think it’s called Truvada”* (LA 25-year-old, Latina, Hispanic, woman). The interviewer follows up by asking her if that was “PrEP” and she responded, *“Yeah. I haven’t used it yet*,* but I’m glad that I have it just in case”*. Later she describes how in the past she has taken medication from Planned Parenthood to treat *“chlamydia and I think it was gonorrhea”*. By making this comparison, it may indicate that this participant did not know that PrEP was intended to be taken prior HIV exposure rather than after, like other medications for sexually transmitted infections she had taken.

#### Interest in/ readiness for future PrEP use

Ambivalence to PrEP was noted by some participants who described engaging in high-risk behaviors. While some participants described a present state of uncertainty in whether to choose to use PrEP, many also expressed contemplation or interest in starting PrEP in the future. For some participants, they expressed that they knew PrEP was something they “should” use, for example:

“I should have took advantage— I should have took advantage and I should have went through the treatment [PrEP]. I still need it, you know.” (Los Angeles 48-year-old, mixed race, Hispanic, man).

Others described PrEP ambivalence based on information obtain through social networks. One participant who started PrEP decided to stop based on her friend’s negative experience with PrEP:

“I got the prescription [PrEP], but when I stared reading more about it and a friend of mine, he took his— he has HIV, but his girlfriend, she doesn’t and they had prescribed it to her – I don’t remember what it was, but she didn’t like it. At first, I was okay with taking it and then I guess listening to my friend, it changed.” (Houston 40-year-old, white, Hispanic, woman).

The reasons participants gave for speculative PrEP interest mirror the reasons given by participants for using PrEP— they engaged in sexual or drug use behaviours that might put them at risk for HIV transmission, or they wanted to use it ‘just in case.’ For example, when discussing the potential of engaging in transactional sex, one participant identified that there is the possibility that someone could be HIV positive and not tell you:

Participant: “Yeah, I took my HIV test on it, everything is good so far. That’s why I don’t want to continue on these drugs because if you continue one day you might not have the money to get it, and I don’t want to sell my body, doing that to get a drug, no, I don’t want to do that, because you can die very quick and easy but you don’t know what a person might have because they don’t tell you, they won’t when they want it, and they won’t tell you whether they sick or not and it be too late, I’ve gone and had sex with them, I’m risking my own self.”

Interviewer: “Right. Have you thought about taking the medication to prevent you from getting the HIV?”

Participant: “Yes, I would love to have it.” (New York City 57-year-old, Black, woman).

A couple participants in monogamous relationships recognized the uncertainty about their partner’s actions and behavior. This was accompanied by an acknowledgment that from a risk perspective, when having sex, you are also with “*everybody else that they’re with”* (Houston 24-year-old, white/ Hispanic, woman). Another participant said to protect himself from HIV, he considered using PrEP instead of solely relying on his partner’s assurances or actions.

“I’m with somebody now. I’ve been with her for quite some time. But there’s times that we’re homeless, so I’m not saying like we’re together 24 hours a day. […] You’ve been gone 10 hours or whatever. Who knows what you’ve done in ten hours? I only know what you saying to me, what you tell me. […] it’s not that I don’t trust her. I just like to protect myself. So if that medication helps me protect myself.” (New York City 50-year-old, white, Hispanic, man).

## Discussion

From an implementation perspective and in the context of facilitated access to oral PrEP for PWIDs, our qualitative findings underscore that the choice to use PrEP was not a simple task, rather, factors affecting the PrEP decision-making process were linked to whether and/or how participants leveraged PrEP as a resource, contingent upon their circumstances. Autonomy, or freedom in decision-making to choose for oneself whether and when to use PrEP, was bounded by structural factors in the external environment, such as unstable housing, precarious financial status, and characteristics of the patient related to urgent medical conditions, and prioritizing substance use treatment over starting PrEP [19, 36]. Autonomy is a central principle in harm reduction strategies [37, 38] and has been evidenced as a key factor in interventions promoting well-being [39–41]. Building on previous literature that has identified access as a key barrier for PrEP use among PWID [32, 33, 42], our findings suggest that facilitated access was not sufficient to motivate decisions to use PrEP for some participants. Participants discussions of PrEP underscored competing and pervasive survival needs that must be acknowledged alongside HIV prevention strategies to better understand the fit of PrEP in the lived realities faced by people actively injecting drugs. In this way, PrEP must be part of a more extensive HIV prevention toolkit [43] whereby PrEP can be a single or combination tool depending on PWIDs’ needs and preferences across the social determinants of health.

Our findings parallel other studies with PWID that suggest that low PrEP uptake is associated with low self-perceived HIV risk, and inadequate knowledge about PrEP [44, 45]. Reassuringly, many participants who considered themselves low risk for HIV detailed their engagement in other HIV prevention strategies related to sexual and drug use behaviours, and this engagement presents an opportunity to integrate PrEP education into existing health promotion when working with PWID. Educational contact-based stigma reduction strategies may also help reduce perceptions that PrEP is for gay men and/or PWID who are ‘dirty’ or ‘not like me’ and may further increase receptivity to using PrEP [44, 45]. Our findings contribute to the existing literature by demonstrating the connection between decision-making power and autonomy, underscoring the need to center the complex lives of PWID in how HIV prevention strategies are implemented. This suggests that by embracing a strength-based approach whereby PrEP education is tailored to PWID, emphasis can be placed on informed decision-making that centers choice amid their HIV prevention options to increase utilization of PrEP.

Similar to previous research, integrated and mobile care was a facilitator of PrEP access and utilization among some PWID [20, 46]. For those participants assigned to the intervention condition, they expressed that mobile health delivery units increased access and privacy, improving their overall healthcare experience. However, external structural factors such as unstable housing were often prioritized over starting PrEP for some individuals. While integrated MOUD and HIV services in brick-and-mortar settings alongside mobile delivery are urgently needed for PWID, comprehensive social services are also necessary to support basic living needs, enhancing structural stability and further supporting harm reduction and HIV prevention interventions. Integrating referrals into community services could address the intertwining health threats related to drug use in addition to infectious disease risks impacting PWID. This may include healthcare provider-facilitated linkage to non-medical social services to support people in addressing housing and economic needs to improve their health and wellness [47, 48].

We identified significant gaps in some participants’ knowledge around PrEP and HIV prevention, including misconceptions about the target population for PrEP, perceiving oneself as low risk for HIV transmission despite acknowledging that they engage in behaviors that increase their vulnerability to HIV, and lack of PrEP literacy as a preventive medication for HIV-negative individuals. These misunderstandings and knowledge gaps may contribute to their reluctance or ambivalence to consider PrEP as a preventive strategy that is relevant to them. While individuals’ perceptions of risk may not always align with their demonstrated risk [49, 50], greater attention to patient-centered narratives can help illuminate low self-perceived HIV vulnerability among PWID. Despite recent studies highlighting willingness to use LAI PrEP among PWIDs [51, 52], our study points to the need to consider a constellation of factors to support uptake. Although directly related to oral PrEP, these findings may be useful in informing this population’s readiness and implementation of long-acting injectable (LAI) PrEP. Addressing issues of autonomy, risk perception, and social determinants of health will be crucial for success across different PrEP modalities. Evidence-based tools such as brief motivational interviewing [53] may be well positioned to help PWID resolve ambivalence and reinforce autonomy when deciding if or when to use PrEP, oral and LAI. Tailored PrEP education for PWID, addressing these misconceptions, can be instrumental in increasing knowledge and self-efficacy. However, it is essential to recognize the heterogeneity within substance use and HIV-affected communities, necessitating an intersectional approach to HIV prevention strategies.

### Limitations

Despite the valuable insights gained from our study, certain limitations should be acknowledged. The qualitative interviews were not designed to assess experiences with PrEP directly, and data collected were limited by what the participant chose to share in the context of the broader implementation science research. For example, the findings presented are not cross-referenced with the RCT clinical treatment records to confirm non-PrEP use among those who stated they were not taking PrEP; rather, quotes seek to illustrate the complex and nuanced reasons for PrEP utilization. Our qualitative findings reflect observations related to implementation determinants on perceptions of evidence-based HIV prevention strategies, and not HPTN 094 clinical outcomes. Further, the sampling procedure was not intended to yield a representative sample of the overall HPTN 094 study. While all participants had facilitated access to PrEP and peer navigation in both study arms, those in the intervention arm may have received more personalized services via more continuity of clinicians and other factors such as the convenience of local location through mobile van healthcare delivery. However, the IS qualitative interviews were not linked to HPTN 094 study arm in the implementation phase, limiting our ability to identify which interview participants were in the intervention and control groups. The interviewed subset of INTEGRA participants may not fully represent all participants’ diverse experiences and perspectives. Geographic heterogeneity is a strength of this HPTN 094 INTEGRA, as participants were recruited from five different geographic sites. While interviews captured varying social, cultural, and contextual factors, participant responses and viewpoints on PrEP could have been influenced accordingly. Additionally, the cross-sectional nature of the IS interviews only captures a snapshot of participants’ experiences and lacks insights into changes over time. Longitudinal qualitative studies could explore evolving attitudes and behaviors regarding PrEP access and utilization among PWID.

## Conclusions

Facilitated PrEP access alone was insufficient to motivate decisions to use PrEP among all PWID interviewed in this qualitative study. The decision to use PrEP was complex, linked to autonomy, risk perception, and pervasive structural vulnerabilities that shaped decision-making around which HIV prevention efforts to use and when. Participants described numerous enacted HIV prevention strategies highlighting that PrEP should be promoted as part of a comprehensive HIV prevention toolkit, allowing for individualized approaches based on user needs and preferences. By recognizing the significance of autonomy in PrEP decision-making, patient-centered interventions can be tailored to better support modifiable factors (i.e., linkages between patient characteristics, external environment, and perceived effectiveness/need for the intervention) in addition to integrated care access to facilitate utilization of HIV prevention strategies among PWID.

## Data Availability

The qualitative data analyzed in the current study is not publicly available. Pseudonymized data are available from the corresponding author on request.

## Abbreviations

HPTN: HIV Prevention Trials Network
MOUD: medications for opioid use disorder
OUD: opioid use disorder
PWID: people who inject drugs
PrEP: Pre-exposure prophylaxis
